# *TXM-Wizard*: a program for advanced data collection and evaluation in full-field transmission X-ray microscopy

**DOI:** 10.1107/S0909049511049144

**Published:** 2012-01-05

**Authors:** Yijin Liu, Florian Meirer, Phillip A. Williams, Junyue Wang, Joy C. Andrews, Piero Pianetta

**Affiliations:** a Stanford Synchrotron Radiation Lightsource, SLAC National Accelerator Laboratory, 2575 Sand Hill Road, Menlo Park, CA 94025, USA; b MiNALab, CMM-irst, Fondazione Bruno Kessler, Via Sommarive 18, Povo, Trento 38123, Italy; c Advanced Photon Source, Argonne National Laboratory, 9700 South Cass Avenue, Argonne, IL 60439, USA

**Keywords:** X-ray microscopy, full-field, tomography, XANES imaging

## Abstract

Transmission X-ray microscopy (TXM) has been well recognized as a powerful tool for non-destructive investigation of the three-dimensional inner structure of a sample with spatial resolution down to a few tens of nanometers, especially when combined with synchrotron radiation sources. Recent developments of this technique have presented a need for new tools for both system control and data analysis. Here a software package developed in MATLAB for script command generation and analysis of TXM data is presented. The first toolkit, the script generator, allows automating complex experimental tasks which involve up to several thousand motor movements. The second package was designed to accomplish computationally intense tasks such as data processing of mosaic and mosaic tomography datasets; dual-energy contrast imaging, where data are recorded above and below a specific X-ray absorption edge; and TXM X-ray absorption near-edge structure imaging datasets. Furthermore, analytical and iterative tomography reconstruction algorithms were implemented. The compiled software package is freely available.

## Abstract 2

A suite of GUI programs written in MATLAB for advanced data collection and analysis of full-field transmission X-ray microscopy data including mosaic imaging, tomography and XANES imaging is presented.

## 1. Introduction

Full-field transmission X-ray microscopy (TXM) using both synchrotron and tabletop X-ray sources has been accomplished at many facilities all over the world (Schneider, 1998 ▶; Chao *et al.*, 2005 ▶; Yin *et al.*, 2006 ▶; Sakdinawat & Liu, 2007 ▶; Takman *et al.*, 2007 ▶; Chu *et al.*, 2008 ▶; de Jonge *et al.*, 2008 ▶; Kim *et al.*, 2008 ▶; Chen *et al.*, 2008 ▶; Andrews *et al.*, 2010 ▶; Rehbein *et al.*, 2009 ▶). The unique capability of imaging three-dimensional structure with spatial resolution down to a few tens of nanometers (Chao *et al.*, 2005 ▶; Schneider *et al.*, 2010 ▶; Liu *et al.*, 2011 ▶) over a field of view of several tens of micrometers has made TXM a well recognized tool for research ranging from materials science (Chen *et al.*, 2008 ▶; Li *et al.*, 2009 ▶; Nelson *et al.*, 2011 ▶) to biological studies (Larabell & Le Gros, 2004 ▶; Andrews *et al.*, 2010 ▶; Patty *et al.*, 2009 ▶; Schneider *et al.*, 2010 ▶).

The rapid development of new experimental applications, which explore the full capability of synchrotron-based TXM, has led to a variety of demands to system control and data evaluation. Two main factors responsible for the variety of imaging techniques, (i) imaging speed and (ii) energy tunability, are made possible through the use of synchrotron radiation sources. The first enables, for example, two-dimensional imaging of large areas by stitching together multiple fields of view, or the combination of time-consuming measurements (such as averaged imaging and imaging at different energies) with tomography. The second, the ability to tune the energy of the incident photons with high resolution, opens the vast field of applications of X-ray absorption spectroscopic (XAS) imaging. XAS imaging applications range from dual-energy (2E) contrast imaging techniques (Yin *et al.*, 2006 ▶; Grew *et al.*, 2010 ▶), in which images directly below and above the absorption edge of a specific element of interest are recorded, to full-field TXM XANES imaging (Meirer *et al.*, 2011 ▶), in which a stack of images is recorded at different energies, generating an absorption spectrum for each pixel within the field of view. The combination of both imaging speed and energy tunability poses a challenge to data handling and processing, and requires automated instrumental control. Hardware and software used for data handling and processing must be optimized for handling large amounts of data and computationally intense tasks. Automated control systems must reliably perform a series of complex movements of optics and sample stage *via* command scripts, in order to fulfil the demands of modern TXM experiments.

In order to meet these requirements we have developed a collection of programs in MATLAB which utilize graphical user interfaces (GUIs) to generate specific command scripts to perform a variety of different TXM experiments, and to process and evaluate data. In this report we present the main features of the five toolkits available within the *TXM-Wizard* software package: (i) script generators for mosaic tomography and TXM-XANES, *i.e.* programs for generating command script files for different experimental set-ups including mosaic image collection, tomographic data acquisition, and imaging experiments involving X-ray energy scans; (ii) 2E imaging toolkit, a program for analyzing differences in contrast of images taken above and below a specific X-ray absorption edge of an element of interest in the sample; (iii) analytical and iterative tomographic reconstructor, a program for tomographic reconstruction of TXM data with options for different algorithms; (iv) mosaic image stitcher, a program that stitches image tiles (multiple fields of view) using auto image alignment and edge-effect correction; and (v) TXM-XANES wizard, a program for processing and analyzing TXM image stacks recorded during an XANES energy scan. Options for performing parallel computing have been implemented in most of these toolkits in order to make full use of multiple-core processors where available. At the time of this writing, *TXM-Wizard* software has more than 380 downloads from over ten countries according to the statistic data from SourceForge.net, and has been used by beamline scientists at different synchrotron facilities including Stanford Synchrotron Radiation Lightsource (SSRL), Advanced Photon Source, National Synchrotron Light Source, Beijing Synchrotron Radiation Laboratory, and National Synchrotron Radiation Laboratory.

## 2. Features of the mosaic tomography and TXM-XANES script generators

Currently, the standard control program for several of the reported TXM systems (Chu *et al.*, 2008 ▶; Chen *et al.*, 2008 ▶; Andrews *et al.*, 2010 ▶) is commercial software (a.k.a. TXMController or XMController) developed by Xradia Inc. XMController provides a non-interactive command-line interface capable of processing script files as well as a GUI for controlling the TXM system. For more efficient manipulation of the system in various complex experimental set-ups, well defined command-line script files must be used. The mosaic tomography and TXM-XANES script generators can be used to generate command-line scripts in order to accomplish these experiments. It is useful to note that the script generators in the current release of *TXM-Wizard* are designed for the Xradia TXM systems. However, given more information about the hardware configuration of other X-ray microscopy systems, the script generators can be easily modified in order to implement command-script-based data acquisition, which is usually more efficient than GUI-based interactive data collection.

### 2.1. Mosaic imaging

One of the most common requirements for the TXM system is to perform two-dimensional mapping and/or tomography over an area that is larger than the available field of view. The script generator allows the user to define the area of interest, and generates a script to perform raster scanning of the sample with appropriate motor movements. In order to overcome possible motor errors, a certain amount of overlap in the raster scan can be defined, enabling post-measurement fine alignment among single image tiles when stitching them together (see §5). Different strategies for reference image collection can be set up in order to optimize data collection speed and quality of reference correction. Mosaic image collection over the same area can be easily repeated within the script (user-defined number of repetitions), for example to achieve better signal-to-noise (S/N) ratio and/or for monitoring of time-dependent processes (*e.g. in situ* monitoring).

### 2.2. XANES imaging

Another strong demand for synchrotron-based TXM systems is to collect a set of images across the X-ray absorption edge of a particular element. X-ray absorption near-edge structure (XANES) analysis is widely used in transmission or fluorescence mode for studying bulk material, and in fluorescence mode with scanning microprobes for better spatial resolution. In the latter case the final image is built from point-by-point information collected *via* raster scanning of the sample using a focused X-ray beam (*e.g.* de Groot *et al.*, 2010 ▶). However, when a full-field TXM system is used for XAS, the experiment and data analysis are quite different, mainly because spectra are extracted from the image stack collected, usually resulting in ∼1 × 10^6^ generated spectra (see §6 and Meirer *et al.*, 2011 ▶). The sample area covered by the field of view (FOV) of the TXM (tens of micrometers) is usually much larger than the area which can be covered by a scanning probe (within reasonable measuring times) when using a beam size offering a comparable spatial resolution (tens of nanometers; the resolution of the TXM).

For XANES imaging, the script generator allows the user to define the range and steps of the desired energy scan, and calculates corresponding zone plate coordinates for each energy point based on the initial inputs. When executing the script the incident X-ray energy is changed and the zone plate is moved simultaneously in order to keep the sample in focus during the scan (see §3). We have also implemented an option to perform an energy scan at different angles of the sample stage, extending this method to XANES tomography (Meirer *et al.*, 2011 ▶). The collection of three-dimensional XANES data can easily be set up within the script generator using two different modes: collecting an energy scan at each angle, or performing tomography at each energy.

## 3. Features of the 2E imaging toolkit

Images taken above and below characteristic X-ray absorption edges can be used to determine the distribution of the corresponding elements within a sample (Grew *et al.*, 2010 ▶). A command-line script to acquire images at multiple energies for these experiments can be easily set up using the script generator described in the previous section. Data evaluation for this experiment requires multiple pre-treatment steps including magnification correction and auto image alignment. The first is necessary because the focal length of the objective Fresnel zone plate is a function of the incident X-ray energy [as given in equation (1)], which leads to the necessity of zone plate movement along the beam axis for focusing the sample,
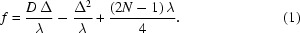
[In (1), *D* is the diameter of the Fresnel zone plate; Δ is the outermost zone width of the zone plate; λ is the wavelength of the incident X-rays; and *N* is the total number of zones.] As a result, the magnification factor varies as a function of the illuminating X-ray energy. Bicubic interpolation is employed to correct the change in magnification in order to obtain consistent image size.

Automatic image alignment is necessary, because both misalignment of the zone plate stage and motor movement errors will cause random relative shifts between images recorded at different energies. Therefore, a phase correlation algorithm (Reddy & Chatterji, 1996 ▶) has been implemented for auto image alignment, which can be used to align all magnification-corrected images of the energy stack before further processing. For 2E difference imaging, the image taken at lower energy (below the absorption edge of a specific element of interest) is subtracted from the one recorded at higher energy (above the edge).

**Figure 1:**
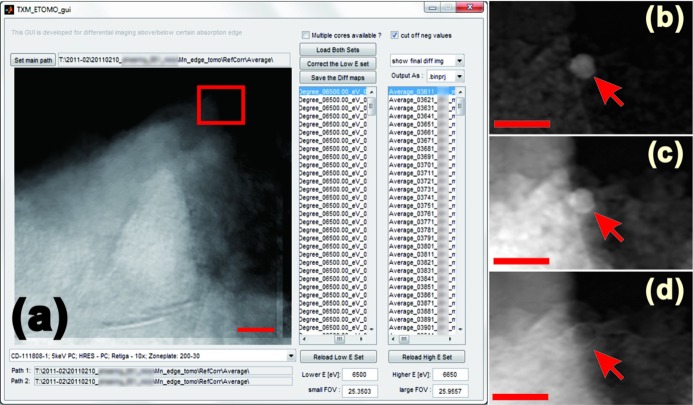
The 2E imaging GUI with a test dataset (a porous Mn-based sample labeled with Au particle) loaded. Panel (*b*) is a magnified view of the highlighted area of the transmission image taken at 6500 eV (below the Mn edge); panel (*c*) is the corresponding view of the image taken at 6650 eV (above the Mn edge); and panel (*d*) is the difference map generated by the program. The scale bar in panel (*a*) is 2 µm, and those in panels (*b*), (*c*) and (*d*) are 0.6 µm.

A screen shot of the GUI for the 2E imaging toolkit is shown in Fig. 1 ▶. A test dataset of a porous Mn-based compound labeled by an Au marker demonstrates the capability of this program. As shown in Figs. 1(*b*) and 1(*c*) ▶, the Au marker is visible in images taken above and below the Mn edge. However, after image correction and subtraction, the contrast of the Au marker is significantly reduced in the difference map owing to the fact that the difference in absorption coefficient of Au over this energy range (6500 eV to 6650 eV) is very small [the change in transmission for a 1 µm Au particle (density 19.32 g cm^−3^) at these energies is ∼2%; see Henke *et al.* (1993 ▶)]. Because this is true over this energy range for all other elements except Mn, which shows an absorption edge jump in this energy region, the remaining enhanced portion of the difference image displayed in Fig. 1(*d*) ▶ shows the Mn distribution in the sample. These difference maps can be generated for entire tomography datasets collected at two energy levels. The output files of the 2E imaging toolkit can be input into the tomographic reconstructor, which will be described in the next section.

## 4. Analytical and iterative tomographic reconstructor

**Figure 2:**
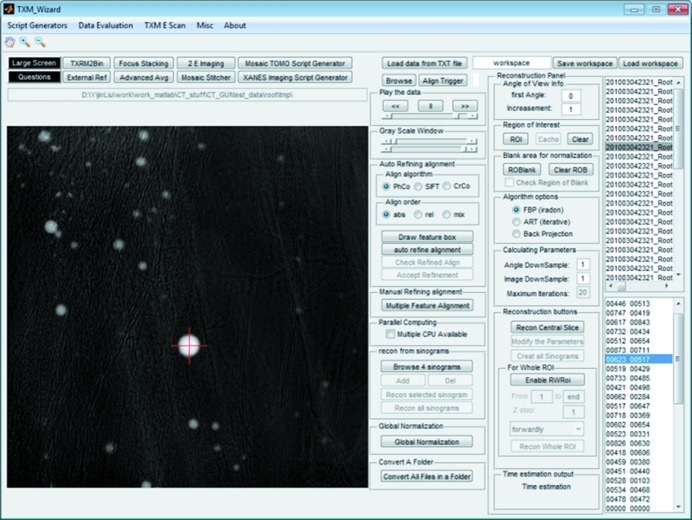
Main window of the analytical and iterative tomographic reconstructor.

Tomography is routinely used in X-ray and electron microscopy for investigation of the three-dimensional inner structure of the sample. Different types of tomographic reconstruction algorithms, including filtered back projection (FBP) (Deans, 1983 ▶) and iterative algebraic reconstruction technique (i-ART) (Shepp & Vardi, 1982 ▶), are available. The reconstructor provides options for using both FBP and i-ART algorithms for tomographic reconstruction. A screen shot of the reconstructor with a test dataset loaded is shown in Fig. 2 ▶.

Considering the spatial resolution of the TXM system (down to a few tens of nanometers depending on the configuration of the Fresnel zone plates), the random shift of the images acquired at different angles, mainly caused by motor jitter, must be corrected prior to tomographic reconstruction. This motor error correction can be handled within the toolkit by image alignment using methods such as manual feature tracking, manual multiple-feature alignment, auto feature tracking and auto alignment refining [based on a phase correlation algorithm, cross correlation algorithm and/or scale-invariant feature transform algorithm (see Vedaldi & Fulkerson, 2008 ▶)]. The region of interest can be easily defined for local tomographic reconstruction, to reduce the size of the output file and subsequently speed up the reconstruction.

It is useful to note that there are several popular software packages, such as *TomoJ* (Messaoudii *et al.*, 2007 ▶), *XMIPP* (Sorzano *et al.*, 2004 ▶), *etc.*, offering various algorithms for tomographic reconstruction from standard transmission data. On the other hand, *TXM-Wizard* is designed to handle data from different X-ray microscopy systems such as diffraction-enhanced phase contrast imaging (Wang *et al.*, 2006 ▶) and grating-based X-ray phase contrast imaging (Zhu *et al.*, 2010 ▶). (Both probe the partial derivative of the refractive index.) In these cases, modified algorithms (Huang *et al.*, 2006 ▶; Liu *et al.*, 2007 ▶) are implemented.

## 5. Mosaic image stitcher

**Figure 3:**
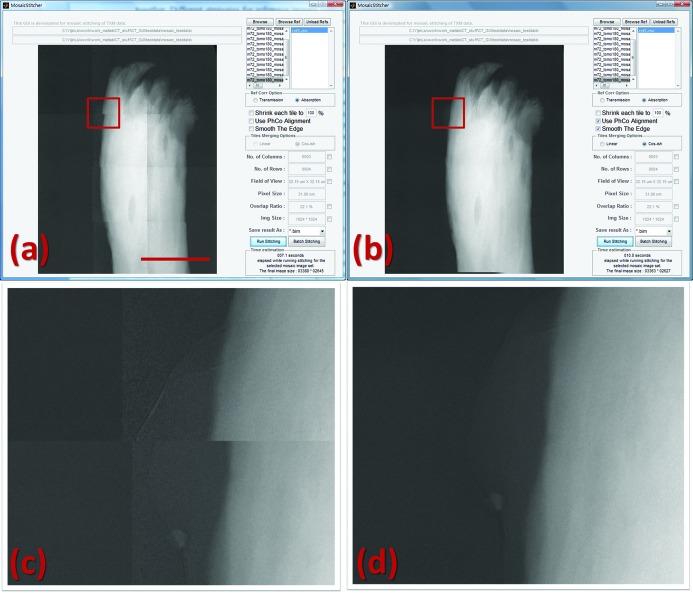
Screen shots of the mosaic image stitcher. Panel (*a*) shows the stitched image using raw motor coordinates; panel (*b*) shows the software-corrected full image; (*c*) and (*d*) are magnified views of the highlighted area in panels (*a*) and (*b*), respectively, visualizing the edge-effect correction. The scale bar in panel (*a*) is 16 µm.

Raster scans with user-defined overlap area can be set up using the script generator described in §2. The mosaic image stitcher was developed for merging overlapping image tiles in order to generate a full image with larger FOV. Motor errors are corrected with phase correlation auto alignment using the overlapping area between tiles. The edges of image tiles can be corrected using a weighted average over the overlapping area. This feature helps to correct an artifact related to the fact that the corners of the images have a poorer S/N ratio than the center of the images. Screen shots of the mosaic image stitcher are shown in Fig. 3 ▶ in which a test dataset is used to demonstrate the capability of the stitcher. In panel (*a*) the image tiles are stitched together using the recorded motor positions, whereas panel (*b*) shows the full image after the procedures described above. Comparing with other programs commercially/freely available for image stitching such as *Photoshop* and *ImageJ*, one main advantage of using the *TXM-Wizard* mosaic image stitcher is the option to make use of the encoded motor positions of each image tile for the initial stitching configuration. When the encoded coordinates are available in the data files the efficiency, speed and success rate of the stitching are improved significantly.

Different output file formats can be used as input to the tomographic reconstructor and/or our image analysis programs related to energy scans (*e.g.* the 2E imaging toolkit and the TXM XANES wizard), including common image formats (*e.g.* JPG, TIF) and binary file formats.

## 6. TXM XANES wizard

**Figure 4:**
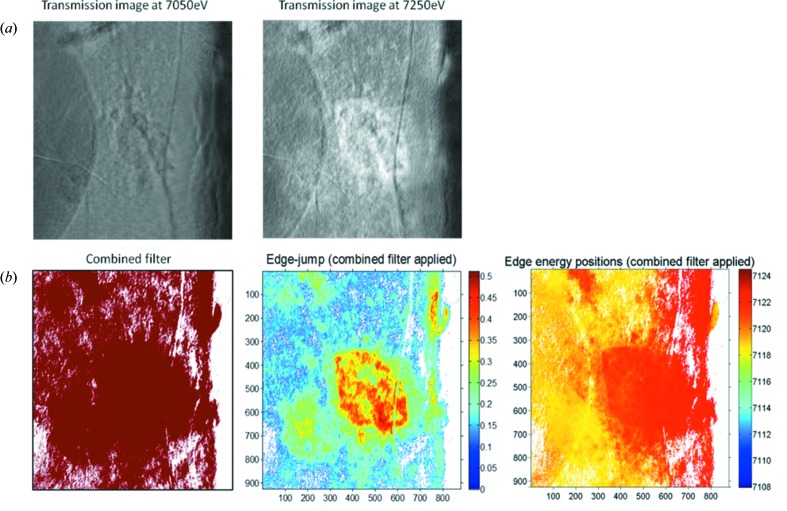
TXM XANES wizard filtering, edge-jump determination and edge-energy determination as performed on a cross section of iron-rich Roman pottery (Sciau *et al.*, 2011 ▶). (*a*) Transmission images recorded below and above the Fe *K*-edge after alignment and magnification correction. (*b*) Left: ‘Combined filter’, a binary image (red = 1, white = 0) showing the applied filter based on edge-jump and normalization filtering (see §6.1 and §6.2 for details). Center: ‘Edge-jump map’, an image showing the magnitude of the Fe *K*-edge jump for each (non-filtered) pixel. This map shows Fe distribution and concentration differences within the analyzed FOV (see §6.1 for details). Right: map of the ‘Edge energy position’, determined as the position of the half-height (0.5) of the normalized edge (see §6.2 for details). For this sample the map shows (at least) two regions of different Fe valence states within the FOV, where the red area has a higher oxidation state than the yellow area.

As mentioned in §2, TXM imaging with energy scans is highly desirable, as it couples high-resolution imaging with X-ray absorption spectroscopy. The main difference between TXM XANES imaging (Fig. 4 ▶) and 2E imaging (Fig. 1 ▶) is that in XANES imaging multiple images are recorded while tuning the incident energy across the absorption edge of a specific element of interest. In this case the goal is not to record differences of the absorption coefficient of two different elements but the modulation of the absorption coefficient of a specific element owing to its chemical state. It is therefore necessary to tune the energy with high resolution (typical step sizes are well below 1 eV). This type of experiment can be set up using the script generator described in §2.

In a standard XAS experiment the beam intensity at the sample must be normalized, which is usually achieved using the flux recorded in the first ionization chamber located between sample and source. In TXM XANES this is routinely achieved by recording and applying a reference image (without sample) at each energy of the XANES scan. After reference correction, magnification correction and auto image alignment for the image stack collected during the energy scan, XAS analysis can be applied to each single pixel within the FOV. A typical FOV contains 1024 × 1024 pixels, resulting in up to 1 × 10^6^ XANES spectra to be processed. Spectroscopic images consisting of such large numbers of spectra are unprecedented, and manual processing of single XANES spectra (as commonly used for bulk XANES) is no longer feasible. Extraction of scientifically meaningful information from these large datasets is a computational challenge, requiring fast and robust procedures for data processing. The TXM XANES wizard provides a series of automated processing options, briefly described in the following sections.

### 6.1. Determination of edge-jump and noise filtering

In the first step for XANES imaging analysis, pixels showing weak absorption (*i.e.* poor S/N ratios of the XANES) can be filtered using the absorption edge jump, defined as the difference between the average intensity value in the post-edge region and the average intensity value in the pre-edge region. Both energy regions are defined by the user. Pixels with edge jumps smaller than a certain threshold are removed (set to zero intensity at all energies). The threshold is calculated for each pixel as the pre-edge standard deviation of that pixel (representing the XANES noise level) multiplied by a user-defined factor. Relevant information about the samples is obtained during this step because the magnitude of the edge jump is proportional to the concentration of the element of interest (Fig. 4*b* ▶).

### 6.2. Normalization and determination of edge energy

Because of the large number of pixels and the fact that some single-pixel XANES can show relatively high noise levels (when compared with bulk techniques), rapid, effective and automated normalization of each XANES is required. Furthermore, because all XANES are recorded during the same energy scan, differences between XANES of different pixels (*e.g.* distortions in the pre- and/or post-edge region owing to different slopes) are exclusively caused by the sample itself. Therefore the normalization procedure uses simple linear regression in the pre- and the post-edge region to normalize the XANES to 0 (pre-edge) and 1 (post-edge). Automated normalization must be capable of processing up to 1 × 10^6^ spectra within reasonable times, and cannot account for single ‘problematic’ pixels for which a more sophisticated manual normalization could be necessary. Therefore, pixels which could not be normalized sufficiently by the algorithm (*i.e.* pixels in which the calculated pre- or post-edge lines show very high or very small slopes) are filtered (set to zero intensity at all energies). The filter threshold for unreasonable slope values can be adjusted by the user. Usually, if the threshold for noise filtering was chosen reasonably well, the number of additionally filtered pixels owing to poor normalization is much less than 1%.

After normalization and normalization filtering, the energy position of the absorption edge can be determined using an approach (Koningsberger *et al.*, 2000 ▶) which is more robust against noise than using the derivative of the XANES: the energy position is interpolated (linearly) for each of the filtered pixels as the energy where the absorption values of the XANES are half the edge-jump intensity (as defined in §6.1). The XANES edge position contains valuable information about, for example, the valence state of the element of interest, and a map of the edge jump therefore provides initial information about the chemistry of the sample (Fig. 4 ▶).

### 6.3. Single-pixel XANES, bulk XANES and analysis of edge-energy distribution

**Figure 5:**
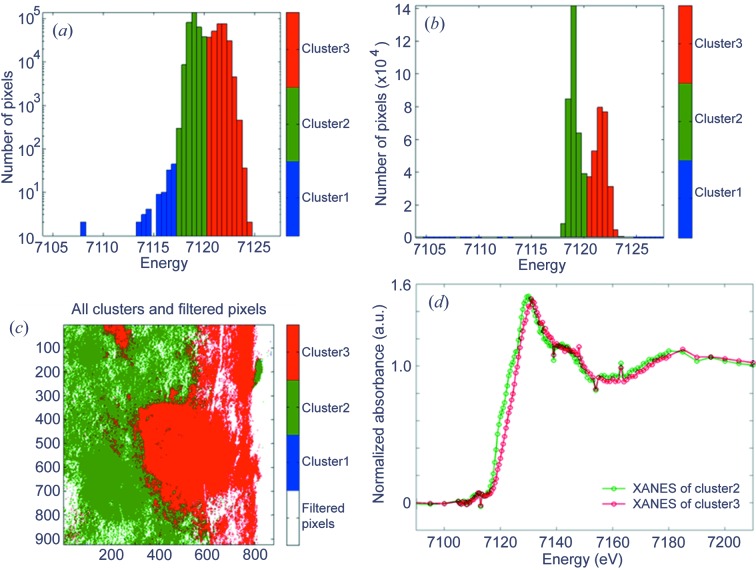
TXM XANES wizard analysis of the edge energy distribution as described in §6.3, performed for the sample introduced in Fig. 4 ▶. The histogram of edge energies *versus* pixel number for all pixels within the FOV is displayed using (*a*) logarithmic scale and (*b*) linear scale and was used to manually pool pixels with similar XANES edge-energy positions. The distribution of the clustered pixels within the FOV is shown in (*c*). The normalized sums of the XANES of all pixels of the same cluster are shown in (*d*). The XANES of the blue cluster is not displayed here as this cluster contains only a few pixels showing very low edge energies. These pixels have been classified as outliers.

At each stage of the analysis of the XANES dataset (*i.e.* with or without any of the above-mentioned filters applied) it is possible to retrieve and display the XANES of a single pixel in the image. Furthermore, the average XANES of all pixels (with or without using filters) can be displayed, which is equivalent to the bulk XANES recorded for the whole FOV (typically 15 µm × 15 µm). However, to find subtle differences in chemistry within the analyzed area it is necessary to cluster pixels with similar XANES. The simplest way to do this is with clustering based on the edge energy distribution in the image: the program offers an interface displaying a histogram of edge energies *versus* pixel number (Fig. 5 ▶). The user can now pool pixels with the same edge energy (within experimental precision, *i.e.* energy step size) or similar edge energies by selecting one or more specific columns in the histogram. Based on this selection a color is assigned to each of these pixel groups to highlight their distribution within the FOV (Fig. 5*c* ▶).

### 6.4. Least-squares linear combination fitting

**Figure 6:**
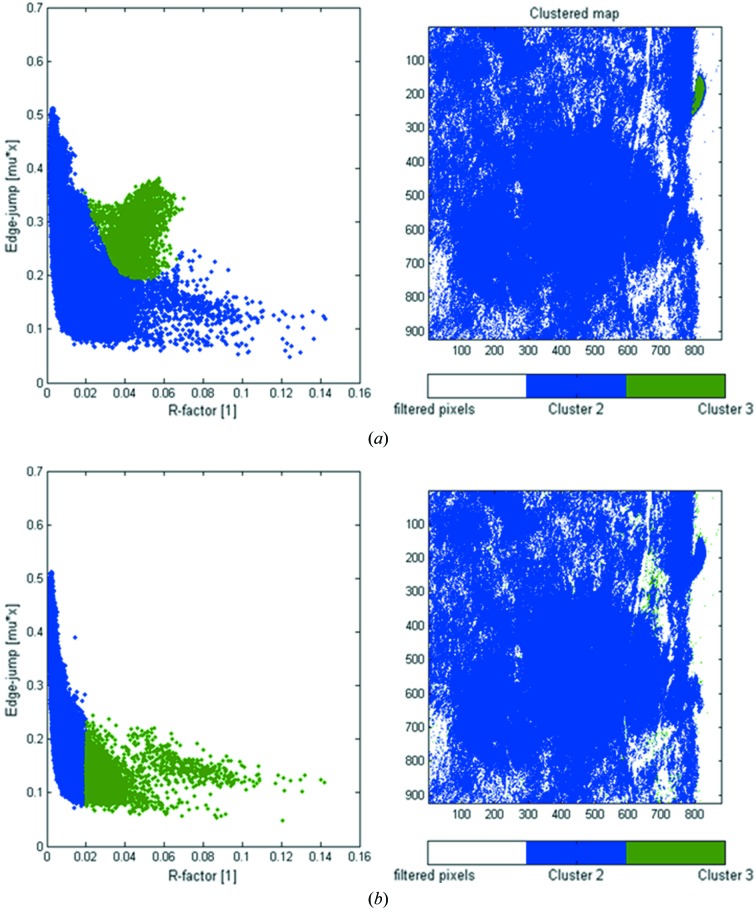
Plots of the edge-jump magnitude *versus* the quality-of-fit parameter *R*-factor determined for each pixel (left), and corresponding cluster maps (right). In (*a*) the XANES of each pixel recorded for the sample introduced in Fig. 4 ▶ have been fit using only two XANES standards. It can be seen that a number of pixels show sufficiently high edge-jump values but poor (large) *R*-factors. After grouping these pixels (*a*, left) by drawing an area of interest in the plot, their distribution is highlighted within the FOV (*a*, right). These pixels cluster in the small area in the right upper corner, suggesting a third Fe phase present there. (*b*, left) The same plot as in (*a*) but using three standards; here the correlation between edge-jump magnitude and *R*-factor behaves as expected: a higher edge-jump value results in a lower *R*-factor (better fit) and *vice versa*. Pixels with *R*-factors > 0.02 are distributed in areas of low Fe concentration [*b*, right; compared with the edge-jump map in Fig. 4(*b*) ▶].

To obtain information about the distribution of the chemical phases of the element of interest present in the sample, least-squares linear combination (LC) fitting of each single-pixel XANES can be performed. This step requires *a priori* knowledge of the phases present in the sample. Future releases of TXM XANES Wizard will include a toolkit for principal component analysis (currently being tested) which will help experienced users find unknown or missing phases in the dataset prior to LC fitting. During LC fitting the loaded set of normalized reference spectra is fit to the XANES of each pixel using a least-squares method. The quality of each LC fit can be checked with the *R*-factor (Ravel & Newville, 2005 ▶) commonly used for XANES fitting, defined as
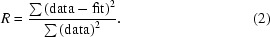
Because one *R*-factor is available for each pixel, an *R*-factor map can be generated to highlight areas of bad fit results (Fig. 6 ▶).

Furthermore, the *R*-factor of each pixel can be used to identify problems owing to insufficient or missing references used in the fitting. By plotting the edge-jump value *versus* the *R*-factor of each XANES, pixels can be identified which show sufficiently high concentrations of the element of interest (edge-jump height) but lower quality fit (high *R*-factor) than other pixels with similar edge-jump height. The presence of such pixels can indicate a problem with the fitting model [missing or incorrect XANES reference(s)]. The GUI allows selection of regions in the correlation plot and grouping of pixels within the selected area. As before, a color is assigned to each of these pixel clusters, and pixels of the same cluster can be displayed in the same color within the original FOV.

### 6.5. Generating RGB phase maps from LC fit results

After LC fitting, each pixel is described as a linear combination of the reference XANES. The ratios can be used to generate a red/green/blue (RGB) map of the FOV by assigning the colors red, green and blue to the pure components used in the fit. If more than three references were used in the fit, the user must assign two references to two colors while the rest of the components are summed and represented by the third color. Finally, to account for absorption differences owing to higher or lower elemental concentration at each pixel, the height of the edge jump (determined as described above and further normalized to values between 0 and 1) is used to correct pixel intensities, therefore representing the total elemental absorption in the final image. This final step also accounts for pixels having higher uncertainties in the fit (larger *R*-factors) owing to lower absorption, as it uses their absorption as a weighing function (*i.e.* a transparency value in the final phase map). Because of this weighting, it was found that additional filtering based on large *R*-factor values was unnecessary.

### 6.6. Expansion to mosaic mapping and three dimensions

The above-described method resulting in a phase map for a single FOV can easily be extended to methods involving multiple FOVs, such as mosaic mapping (§5) or tomography. Multiple mosaic tiles of RGB phase maps can be stitched together as explained in §5 to generate a phase map of a large FOV (toolkit not yet included in the base package of the software). Generation of RGB phase maps from each FOV of a tomographic scan (resulting in one phase map for each angle) enables the reconstruction of the three-dimensional distribution of the chemical phases in the analyzed sample volume (toolkit not yet included in the base package of the software). By adding the third dimension, not only chemical speciation but also information about the morphology of the sample is obtained for a given large volume. Furthermore, any overlap of contributions to the attenuation seen in two dimensions is removed during reconstruction, providing the opportunity to probe the sample at different depths. Three-dimensional XANES microscopy using TXM enables chemical speciation of an element of interest within sample volumes which are three orders of magnitude larger (typically 15 × 15 × 15 µm) than the voxel size (typically 15 × 15 × 15 nm). [The three-dimensional resolution is usually estimated to be approximately double the resolution achieved in two dimensions (Cardone *et al.*, 2005 ▶; Schneider *et al.*, 2010 ▶) or about 60–70 nm; determined by the optics of the microscope.] The capability to perform such an analysis within reasonable time frames (hours) is unprecedented and was reported for the first time by Meirer *et al.* (2011 ▶).

## 7. Conclusions and outlook

The *TXM-Wizard* software package was developed for advanced methods in synchrotron-based transmission X-ray microscopy. Advanced TXM methods can be categorized into three basic experimental modes: (i) mosaic mode, in which multiple FOVs are composed to generate one larger FOV; (ii) tomographic mode, using tomographic reconstruction techniques to image the sample in three dimensions; and (iii) energy mode, in which the images are recorded at different incident X-ray energies to provide chemical information. Experiments utilizing one of these advanced methods or combinations of them (*e.g.* mosaic tomography, mosaic XANES, XANES tomography) require a high degree of automation both in system control and data evaluation. *TXM-Wizard* includes user-friendly tools to successfully plan and collect data for these experiments (script generators), pre-process the large number of images recorded (advanced averaging, image alignment, filtering), and evaluate the final datasets (mosaic stitching, three-dimensional reconstruction, elemental imaging, XANES imaging).

Future developments of the software will focus on speed improvement (algorithms, higher degree of parallelization), user-friendly batch modes to process datasets generated by combining advanced methods (*e.g.* XANES tomography), and additional toolkits (*e.g.* principal component analysis of XANES data).

## 8. Resource

A project page for *TXM-Wizard* exists at SourceForge.net, http://sourceforge.net/projects/txm-wizard/. Executable files of *TXM-Wizard* are compiled for windows 32-bit and 64-bit, and both are freely available at the web page mentioned above, as well as a short user’s manual.
